# Risk Factors of Childhood Tuberculosis in the Centre Region of Cameroon: An Unmatched Case–Control Study

**DOI:** 10.1155/ipid/7988364

**Published:** 2025-12-18

**Authors:** Thomas Achombwom Vukugah, Derick Akompab Akoku, Micheline Mekemnang Tchoupa, Edward Lambert

**Affiliations:** ^1^ ICAP in Cameroon, Mailman School of Public Health, Columbia University, Yaounde, Cameroon, columbia.edu; ^2^ Department of Global Health, University of Washington, Seattle, Washington, USA, washington.edu; ^3^ Regional Delegation, Ministry of Public Health, Centre Region, Yaounde, Cameroon, minsante.cm; ^4^ Department of the School of Social and Human Studies, Atlantic International University, Honolulu, Hawaii, USA, aiu.edu

**Keywords:** Cameroon, case–control study, childhood, risk factors, tuberculosis

## Abstract

**Background:**

Even though childhood tuberculosis (TB) is of public health significance in Cameroon, reports on childhood TB and its risk factors are limited. The objective of this study was to identify the risk factors for TB among children in the Centre Region, Cameroon.

**Methods:**

An unmatched case–control study was conducted at ten health facilities in the Centre Region from February 12 to July 31, 2022. Children under 15 years who attended these health facilities for TB treatment were considered as cases. Controls were children attendees who presented in the outpatient department (OPD) of the same health facility for non‐TB health problems. For each case, two consecutive controls were sampled systematically. Data were collected using pretested and structured questionnaires through face‐to‐face interviews with parents/guardians. Logistic regression analyses were employed to identify risk factors for TB.

**Results:**

A total of 120 cases and 231 controls were enrolled in the study, and the median (interquartile range) age among both cases and control was 3 years (IQR = 1–3). The odds of TB were seven times (AOR = 7.24; 95% CI: 1.76–18.98) more likely among children with the absence of BCG vaccination compared with being vaccinated for BCG. A child’s previous history of TB, family history with a TB patient, duration of contact with a TB patient, and child’s HIV status were the other risk factors of childhood TB with AOR 20.01 (4.77–33.87), 4.90 (1.10–21.83), 4.76 (1.02–12.14), and 2.73 (1.55–19.10), respectively.

**Conclusion:**

The absence of BCG vaccination, the child’s previous history of TB, family history with a TB patient, duration of contact with a TB patient, and HIV‐positive status are the independent risk factors for childhood TB in the Centre Region. Contact tracing and contact screening should be enhanced and correctly implemented by the National TB Control Program.

## 1. Introduction

Despite the progress in reducing tuberculosis (TB) incidence and mortality in the last 20 years, TB remains a leading cause of death in children under 5 years [1]. The paucibacillary nature of TB disease and the challenges in collecting expectorated sputum from children continue to make childhood TB significantly underreported and undiagnosed [2–5]. In addition, there are a limited number of frontline healthcare workers who have the capacity and confidence to clinically diagnose childhood TB [6]. Children and young adolescents (aged below 15 years) represent about 11% of all people with TB globally. Every year, 1.1 million children become ill with TB, almost half of them below 5 years of age. In 2021, an estimated 6.7 million cases of all forms of TB were detected globally with 7% (469,000) of the cases diagnosed among children below 15 years of age [7]. TB infection in children leads to TB disease more quickly and frequently than in adults [8]. Childhood TB indicates recent Mycobacterium TB infection, and children with latent infection serve as a reservoir for adult disease [9].

Cameroon is among the top 30 high TB‐ and TB/HIV‐burden countries, and about 20% (4375) of the total number of TB cases were co‐infected with HIV in 2021 [7]. In 2022, an estimated 25,286 cases of all forms of TB were detected across the country with 5% (1264) of the cases diagnosed among children below 15 years of age. This percentage (5%) of children diagnosed with TB during this period was below the expected 10%–12% childhood TB case target set by the country [10].

The Centre Region of Cameroon is one of the four regions in the country with a high burden of TB. In 2022, the Centre, Littoral, North, and Far North regions accounted for 59% of all notified TB cases in the country [11]. In 2022, of the 5260 TB cases diagnosed in the Centre Region, 9% (473/5260) was among children less than 15 years of age [10]. Childhood TB in the country has long been overlooked by clinicians, personnel at the Ministry of Health (MOH), and the general public who believe that children are rarely infectious and thus play a minor role in TB disease transmission [12]. To date, several studies in other countries have investigated the risk factors of childhood TB. Several lines of evidence suggest that contact history, HIV‐positive status, and nutritional status were correlated with childhood TB [13–16]. Other factors associated with childhood TB were nonurban residence, smoking, absence of cross‐ventilation, overcrowding, age, and father’s occupation [17–19].

Although childhood TB is an important public health issue in Cameroon, the factors contributing to the high burden of childhood TB in the Centre Region in particular and Cameroon in general are not yet fully understood [20]. Moreover, health facility data collected and stored in paper‐based registers are rarely analyzed to inform decision‐making at the health facility level. The lack of reliable data makes it difficult for the MOH and international nongovernmental organizations working in the area of childhood TB to tailor interventions designed to reduce the burden of childhood TB in the country. Understanding the risk factors of childhood TB occurrence is crucial to conducting an effective strategy of ending TB as a public health problem. The objective of this study was to identify the risk factors of TB among children in health facilities in the Centre Region of Cameroon. The findings of this study will enable the MOH, the community, and parents to put in place preventive measures to fight against childhood TB.

## 2. Materials and Methods

### 2.1. Study Setting and Period

The Centre Region occupies 69,685 km^2^ of the central plains of the Republic of Cameroon with an estimated population of 5,038,553 (2023) inhabitants. Over the past 6 years, the Centre Region has been overcrowded with internally displaced persons from the North and South West regions. The Centre Region has many health facilities of various categories (e.g., general hospitals, central hospitals, regional hospitals, district hospitals, district medical centers, integrated health centers, and ambulatory health centers), and most of these health facilities are found in Yaoundé (the largest city in the region and the political capital of Cameroon). The Centre Region has 55 TB diagnosis and treatment centers (DTCs) and eight TB treatment centers (TCs). The DTCs are health facilities with specialized laboratory equipment and supplies for TB diagnosis (e.g., Xpert MTB/RIF, smear microscopy, and culture) and treatment, while TCs are health facilities that provide only TB treatment. Therefore, TB diagnosis, care, and management are implemented across 63 health facilities across the region. The study was conducted in health facilities of the Centre Region, from February 12 to July 31, 2022.

### 2.2. Study Design and Population

This study was a facility‐based unmatched case–control study conducted among cases (children under 15 years diagnosed with TB disease) and controls (children under 15 years without TB disease).

### 2.3. Inclusion and Exclusion Criteria

#### 2.3.1. Inclusion Criteria

Children (< 15 years of age) diagnosed with TB disease who attended one of the health facilities for TB treatment were considered as cases. Controls were children (< 15 years of age) attendees who presented in the outpatient department (OPD) at the same health facility as the cases for non‐TB health problems.

#### 2.3.2. Exclusion Criteria

Parents/guardians of children who refused to participate in the study even after consent were excluded. Children (cases or controls) who were critically ill during data collection and those who came to the health facility without their parents/guardians were excluded because they were considered not being able to respond to the study questions. Children with TB who were attending OPD services for non‐TB health problems were excluded because they might have been selected as cases in the TB diagnostic and treatment unit. To avoid bias, children who had TB symptoms (defined as a cough lasting more than 2 weeks, failure to thrive and/or weight loss, night sweating, and cervical and inguinal lymph node swelling) in the last 4 weeks prior to the study were excluded from the cases and controls.

### 2.4. Selection of Health Facilities

Due to time and budgetary constraints, the study team purposively selected ten health facilities in the Centre Region where data had to be collected. The study team aggregated data on the number of children attendees and children diagnosed and initiated on TB treatment in each of the 55 health facilities in the Centre Region during a 3‐year period (January 1, 2018, to December 31, 2020). The team later reviewed the aggregated data (sum) and selected the top ten health facilities with the highest number of children attendees, children diagnosed and initiated on TB treatment during the 3‐year period. These ten health facilities were selected to participate in the study because the study team believed that there would be a higher likelihood of identifying children diagnosed and receiving TB treatment in these facilities based on historic data. In addition, as per the seven main categories in which healthcare facilities are organized in Cameroon, these facilities were within the general, central, or district categories and had the capacity for diagnosis of TB in children (i.e., the capacity to collect sample type, availability of Xpert, and availability of CXR).

#### 2.4.1. Sample Size Calculation for Cases and Controls

The sample size was calculated using a formula for two population proportions for an unmatched case–control study [21] and calculated using Epi Info version 7.2.5 statistical software package by employing the proportion difference approach. The proportion difference approach is used because we assume that there is an exposure difference on factors among cases and controls. To detect whether the assumed difference exists between the two and since there were no study found in the study area and similar population, the following assumptions were used: 1) odds ratio: 2.0, 2) confidence interval: 95%, 3) power of the study: 80%, and 4) the ratio of cases to controls: 1:2. Inputting these data generated a sample size of 411 (i.e., 137 cases and 274 controls). We considered a 5% nonresponse rate, and the total estimated sample size for the study was 432 (i.e., 144 cases and 288 controls).

### 2.5. Sampling Procedure

#### 2.5.1. Selection of Cases

The cases were children (< 15 years of age) with newly bacteriologically confirmed or clinically diagnosed pulmonary TB enrolled for treatment in the selected health facilities. TB diagnosis was made based on the national technical guide for health personnel [20]. The guide is applicable in every part of the country. All newly registered children with TB were considered until the required sample size was reached.

#### 2.5.2. Selection of Controls

Children attendees who presented in the OPD at the same health facility as the cases for non‐TB health problems were selected as control. The ratio of cases vs controls selected was 1: 2. For each case, two consecutive controls that met the inclusion criteria were systematically sampled from the OPDs.

### 2.6. Development of Data Collection Tool

A draft study questionnaire was developed in English after reviewing the published literature [22–24]. The questionnaire was designed to collect data on the sociodemographic, household, and health‐related characteristics of children (cases and controls). The draft questionnaire was shared with five experts (three clinicians and two epidemiologists) with technical knowledge and experience on childhood TB for review and content validation. Their feedback and suggestions guided the revision of the questionnaire. The revised questionnaire in English was then translated into French and back‐translated into English to ensure coherence and consistency. Given that there were some inconsistencies in the translation of a few phrases and instructions, the study team liaised with each other and agreed on the appropriate translations of these phrases from English to French. The English and French versions of the questionnaire (given that the Centre Region of Cameroon is predominantly French‐speaking) were pilot tested among 15 children diagnosed with TB (cases) and 30 children without TB (controls) in two health facilities in Yaoundé that were not selected to participate in the final study.

The pilot study informed the study team about the ease of answering the questions, the wording, the language and understanding of the questions, the number of questions, and the time taken to answer the questions. Based on results and feedback from the pilot testing, relevant changes (e.g., reducing the number of questions and rewording certain questions and instructions), and modifications were made in the final version of the questionnaire used by the study team.

### 2.7. Variables of Interest and Measures

#### 2.7.1. Outcome Variable

The outcome variable was childhood TB (as defined by the cases and controls).

#### 2.7.2. Exposure Variables

The exposure variables were (1) sociodemographic characteristics (age of the child, gender, place of residence, religion, ethnicity, child’s educational status, mother’s educational status, father’s educational status, mother’s occupation, father’s occupation, and monthly family income); (2) household living conditions (household size, number of rooms, number of windows, kitchen location, type of toilet, household waste disposal, source of drinking water, and type of domestic animals); and (3) past medical history of the child (child’s previous history of TB, history of TB among family members, previous contact with a TB patient, duration of contact with a TB patient, previous history of hospitalization, type of chronic disease, child’s HIV status, parent/guardian HIV status, respondent’s relationship with the child and BCG vaccination status assessed based on the presence of a scar on the right upper arm of the child, etc.). These variables were identified after reviewing the published literature [9, 13, 15, 17–19].

### 2.8. Data Collection Procedures

Data collection took place from February 12 to July 31, 2022, by trained TB nurses in each of the ten health facilities that were selected to participate in the study. The study team identified only TB nurses who were bilingual (i.e., proficient and being able to administer the questionnaire in French and English). This was an important consideration since Cameroon is a bilingual country, individuals (in this case parents/guardians of children) who visit a health facility communicate in French and/or English. A one‐day training workshop was organized in collaboration with the MOH, and these TB nurses were trained on the study objectives, the questionnaire, standard operating procedures (SOPs) for recruiting study participants through their parents/guardians, ethical issues including confidentiality and privacy, data storage at the health facility, and data transmission to the study team.

#### 2.8.1. Enrollment of Cases at the Health Facility

In each of the ten selected health facilities, the trained TB nurse was responsible for enrolling the cases and controls. Once a child was diagnosed with TB in any service and sent to the TB department for treatment initiation, the TB nurse enrolled the child for the study exactly the same day of treatment initiation. The same recruitment procedure was applied by the trained TB nurses across the ten health facilities based on the SOP that was developed. The TB nurse approached the parents/guardians to request their participation in the study after explaining the study objectives and ethical considerations. Parents/guardians who expressed interest were provided an informed consent form to sign. The TB nurse then administered the paper‐based questionnaire to the parent/guardian.

#### 2.8.2. Enrollment of Controls at the Health Facility

Once a case was enrolled at a health facility, the TB nurse then contacted the coordinator of the OPD at the same health facility to enroll two controls that met the inclusion criteria. Controls were systematically sampled among children attendees at the OPD. For each enrolled case, two controls were identified in the OPD within a 3‐day period (3 days average patient flow) based on the calculated 3‐day patient flow for each of the health facilities. This approach was applied to increase the chances of identifying and enrolling controls.

For both cases and controls, the TB nurse administered the questionnaire to parents/guardians of these children after confirming their medical diagnosis [i.e., TB diagnosis and on treatment (cases) and non‐TB health problems (control)] by reviewing their patient charts (medical booklet). Data were collected using a validated paper‐based questionnaire in French or English through face‐to‐face interviews with parents/guardians of cases and controls. Past medical conditions were verified by checking the patient medical booklet and based on the information provided by caregivers. BCG vaccination was also physically checked by verification of the scar on the left upper arm, and data on malnutrition were obtained by collecting information on age and height of the patient.

The enrollment of cases and controls continued across all ten selected health facilities for over a 6‐month period to attain the required sample size. At the end of each month during the 6‐month data collection period, all administered questionnaires transferred from the health facility were reviewed by the principal investigator for completeness and then stored in a log file. Where there were issues of data quality, feedback was sent to the TB nurses in their respective health facilities for reconciliation.

### 2.9. Data Analysis

All completed questionnaires were checked, cleaned, coded, and entered into an Excel file. Where there were discrepancies, the data were adjusted and corrected by referring to the questionnaires. The cleaned dataset was then imported into the Statistical Package for Social Sciences (SPSS) software version 24.0 (IBM, Armonk, NY, USA) for analysis.

Descriptive statistics were summarized using frequencies and proportions for categorical variables and median (interquartile range) for continuous variables. The differences in proportions between cases and controls were tested using chi‐square where appropriate; otherwise, Fisher’s exact test was used. The chi‐squared (*χ* ^2^) test was used to identify associations between categorical variables and the study outcomes (cases vs controls). Variables that showed significant differences in sociodemographic and household characteristics and child’s past medical history between cases and controls at a *p* value of < 0.20 in bivariate analysis were considered candidates for logistic regression analyses.

Unconditional multivariable logistic regression analysis was used to determine the independent risk factors for childhood TB, adjusting for confounders. Variables that were not significant at a 5% significance level were removed using a backward stepwise logistic regression model. The Hosmer and Lemeshow goodness‐of‐fit test was used to investigate the goodness of fit. Adjusted odds ratios (AORs) and 95% confidence intervals (CIs) were used to identify the independent risk factors for childhood TB. The level of significance was set at *α* < 0.05.

## 3. Results

The parents/guardians of 432 children were approached to participate in the study. Of these, the parents/guardians of 351 children (120 were cases and 231 were controls) expressed their willingness to participate in the study, which gives an overall response rate of 81.3%. The most common reasons for not participating was lack of time by the caregivers. Due to the incompleteness of the majority (85%) of the data on malnutrition (due to the lack of a stadiometer for height measuring in most facilities, thus not possible to calculate BMI), malnutrition was excluded from the risk factors analysis.

### 3.1. Sociodemographic Characteristics

Table 1 shows the sociodemographic characteristics of the study participants. Of the 351 participants, 120 were cases and 231 were controls. The median age was 3 years (IQR = 1–3) for both cases and control groups, and the majority (50.0%) of cases and 64.9% of controls were within the age group of 0–4 years old. Among the cases, 57 (47.5%) and 63 (52.5%) were males and females, respectively. Among the controls, 121 (52.4%) and 110 (47.6%) were males and females, respectively. More than half of both cases [88 (73.3%)] and control [162 (70.1%)] were from urban residences. Regarding the occupational status of the mother, among cases, 57 (47.5%) were housewives and 9 (7.5%) were employed in the private sector, while among controls, 94 (40.7%) were housewives and 31 (13.4%) were employed in the private sector.

**Table 1:** Sociodemographic characteristics of childhood TB patients and controls in health facilities of Centre Region, Cameroon, 2022.

Characteristics	Cases (*N* = 120)	Control (*N* = 231)	*p* value^1^
	*N* (%)	*N* (%)	
*Age*			0.001
0–4	60 (50.0)	150 (64.9)	
5–9	33 (27.5)	60 (26.0)	
10–14	27 (22.5)	21 (9.1)	

*Sex*			0.386
Female	63 (52.5)	110 (46.6)	
Male	57 (47.5)	121 (52.4)	

*Residence*			0.529
Urban	88 (73.3)	162 (70.1)	
Rural	32 (26.7)	69 (29.9)	

*Religion of the child*			0.304
Catholic	76 (63.3)	127 (55.0)	
Protestant	29 (24.2)	61 (26.4)	
Pentecostal	7 (5.8)	26 (11.3)	
Muslim	8 (6.7)	17 (7.4)	

*Ethnicity*			0.463
Beti	54 (45.0)	88 (38.1)	
Bassa/Mbam	9 (7.5)	30 (13.0)	
Haoussa	6 (5.0)	9 (3.9)	
Bamileke/Grassfield	38 (31.7)	74 (32.0)	
Others^∗^	13 (10.8)	30 (13.0)	

*Child’s educational status*			0.017
Not yet enrolled	50 (41.7)	123 (53.2)	
Nursery	19 (15.8)	39 (16.9)	
Primary	35 (29.2)	58 (25.1)	
Secondary and above	16 (13.3)	11 (4.8)	

*Mother’s educational status*			0.007
Did not attend school	9 (7.5)	13 (5.6)	
Primary	26 (21.7)	23 (10.0)	
Secondary	70 (58.3)	145 (62.8)	
University	15 (12.5)	50 (21.6)	

*Father’s educational status*			0.787
Did not attend school	6 (5.0)	8 (3.5)	
Primary	12 (10.0)	24 (10.4)	
Secondary	75 (62.5)	138 (59.7)	
University	27 (22.5)	61 (26.4)	

*Mother’s occupation*			0.345
Business	36 (30.0)	72 (31.2)	
Civil servant	18 (15.0)	34 (14.7)	
Private sector	9 (7.5)	31 (13.4)	
Housewife	57 (47.5)	94 (40.7)	

*Father’s occupation*			0.026
Business	34 (28.3)	104 (45.0)	
Civil servant	28 (23.3)	40 (17.3)	
Private sector	41 (34.2)	63 (27.3)	
Others^∗∗^	17 (14.2)	24 (10.4)	

*Monthly family income*			0.376
less than 100,000 XAF	31 (25.8)	47 (20.3)	
100,000–250,000 XAF	77 (65.2)	165 (71.4)	
Above 250,000 XAF	12 (10.0)	19 (8.2)	

^∗^Fulfulde, Baya, Littoral.

^∗∗^Driver, security guard, and retired.

^1^ *p* value from the *X* ^2^ test.

### 3.2. Living Conditions

Table 2 shows the living condition of the study participants. Majority of the cases [61 (50.8%)] and 127 (55.0%) of the controls live in households of less than three rooms, and 11 (9.2%) of cases and 14 (6.1%) of controls live in households of more than five rooms. In terms of kitchen location, 55 (45.8%) of cases and 120 (51.9%) of controls live in households with indoor kitchens only, while 9 (7.5%) of cases and 38 (16.5%) of controls live in households with both indoor and outdoor kitchens.

**Table 2:** Living conditions of childhood TB patients and controls in health facilities of Centre Region, Cameroon, 2022.

Variable	Case (*N* = 120)	Control (*N* = 231)	*p* value^1^
	*N* (%)	*N* (%)	
*Household size*			
< 3 members	33 (27.5)	51 (22.1)	0.165
4–6	46 (38.3)	113 (48.9)	
7+	41 (34.2)	67 (29.0)	

*Number of rooms*			
< 3	61 (50.8)	127 (55.0)	0.510
3‐4	48 (40.0)	90 (39.0)	
5+	11 (9.2)	14 (6.1)	

*Number of windows*			
< 3	43 (35.8)	81 (35.0)	0.784
4+	77 (64.2)	150 (65.0)	

*Kitchen location*			
Indoor	55 (45.8)	120 (51.9)	0.006
Outdoor/separated	56 (46.7)	73 (31.6)	
Both	9 (7.5)	38 (16.5)	

*Type of toilet*			
Water closet (modern)	43 (35.8)	82 (35.5)	0.153
Pit toilet (latrine)	66 (55.0)	111 (48.1)	
Both	11 (9.2)	38 (16.5)	

*Household waste disposal*			
Trash can in the compound	73 (60.8)	158 (68.4)	0.156
Trash can in the neighborhood	47 (39.2)	73 (31.6)	

*Source of drinking water*			
Tap water	48 (40.0)	41 (17.7)	< 0.001
Well water	7 (5.8)	10 (4.3)	
Borehole	49 (40.8)	133 (57.6)	
Spring water	11 (9.2)	34 (14.7)	
Bottled water	5 (4.2)	13 (5.6)	

*Type of domestic animals*			
None	78 (65.0)	171 (68.8)	0.282
Dogs	13 (10.8)	14 (6.1)	
Cats	15 (12.5)	28 (12.1)	
Goats	6 (5.0)	6 (2.6)	
Others^∗^	8 (6.7)	24 (10.4)	

^∗^Fowls, pigeons, pigs.

^1^ *p* value from the *X* ^2^ test.

### 3.3. Past Medical History

Table 3 shows that majority of the cases [104 (86.7%)] and controls [223 (96.5%)] had received BCG vaccine. Fifty (41.7%) of the cases had a previous history of TB, whereas only 9 (3.9%) of controls had a previous history of TB. Seventy‐one (55.8%) of the cases and 55 (23.8%) of the controls had a recent family history of TB. Twenty‐three (9.2%) of the cases and 17 (7.3%) of the controls were infected with HIV.

**Table 3:** Past medical history of childhood TB patients and controls in health facilities of Centre Region, Cameroon, 2022.

Variable	Case (*N* = 120)	Control (*N* = 231)	*p* value^1^
	*N* (%)	*N* (%)	
*BCG vaccination status*			0.001
Yes	104 (86.7)	223 (96.5)	
No	16 (13.3)	8 (3.5)	

*Child’s previous history of TB*			< 0.001
Yes	50 (41.7)	9 (3.9)	
No	70 (58.3)	222 (96.1)	

*Family history with a TB patient*			< 0.001
Yes	71 (55.8)	55 (23.8)	
No	49 (44.2)	176 (76.2)	

*Previous contact with a TB patient*			< 0.001
Yes	67 (57.3)	50 (21.6)	
No	53 (22.6)	181 (78.4)	

*Duration of contact with a TB patient*			0.007
1 week	12 (18.8)	19 (38.8)	
2‐3 weeks	20 (31.2)	19 (38.8)	
1–6 months	32 (50.0)	11 (22.4)	

*Previous history of hospitalization*			0.824
Yes	65 (54.2)	128 (55.4)	
No	55 (45.8)	103 (44.6)	

*Type of chronic disease*			0.605
Obesity	2 (1.7)	3 (1.3)	
Asthma	2 (1.7)	8 (3.5)	
Malnutrition	7 (5.8)	11 (4.8)	
Others^∗^	13 (10.8)	16 (6.9)	
None	96 (80.0)	193 (83.5)	

*Child’s HIV status*			0.004
Positive	23 (9.2)	17 (7.3)	
Negative	87 (72.5)	191 (82.7)	
Unknown	10 (8.3)	23 (10.0)	

*Parent/guardian HIV status*			0.033
Positive	23 (19.2)	23 (10.0)	
Negative	89 (74.2)	197 (85.3)	
Unknown	8 (6.7)	11 (4.8)	

*Respondent’s relationship with the child*			0.044
Father	14 (11.7)	27 (11.7)	
Mother	81 (67.5)	151 (65.4)	
Family member	16 (13.3)	48 (20.8)	
Guardian	9 (7.5)	5 (2.2)	

^1^ *p* value from the *X* ^2^ test.

^∗^Cardiopathy, anemia, respiratory infection.

### 3.4. Risk Factors of Childhood TB

Table 4 shows the risk factors of childhood TB. In the univariate analysis, age, child’s educational status, mother’s educational status, father’s occupation, kitchen location, BCG vaccination status, child’s previous history of TB, history of TB among family members, previous contact with TB patients, duration of contact with a TB patient, and child’s HIV status were found to be risk factors of childhood TB. After adjusting for confounders, BCG vaccination status, child’s previous history of TB, history of TB among family members, duration of contact with a TB patient, and child’s HIV status were found to be independent predictors for occurrence of TB in children. Higher odds of TB were associated with the absence of BCG vaccination compared with being vaccinated for the BCG vaccine (AOR = 7.24; 95% CI: 1.76–18.98). Another important variable that shows a strong association was child’s previous history of TB. The odds of TB among those who had previously had TB was 20 times higher compared to those who did not have previous history of TB (AOR = 20.01; 95% CI: 4.77–33.87). Higher odds of childhood TB were also associated with families with TB history. The odds of TB among children who had a recent family history with a TB patient was four times higher compared to those who did not have a family history with a TB patient (AOR = 4.90; 95% CI: 1.10–21.83); those children who had contact with a TB patient for a few months compared to those for only a week were four times more likely at risk of developing TB (AOR = 4.76; CI: 1.02–12.14). In addition, the odds of childhood TB among HIV‐positive children were high compared to HIV‐negative children (AOR = 2.73; CI: 1.55–19.10).

**Table 4:** Logistic regression analysis of risk factors of childhood TB in health facilities of Centre Region, Cameroon.

Characteristics	Case (*N* = 120)	Control (*N* = 231)	Univariate analysis		Multivariate analysis	
	*N* (%)	*N* (%)	COR (95% CI)	*p* value	AOR (95% CI)	*p* value
*Age*						
0–4	60	150	Reference		Reference	
5–9	33	60	1.37 (0.81–2.31)	0.230	1.47 (0.33–6.44)	0.610
10–14	27	21	3.21 (1.68–6.12)	< 0.001	4.61 (0.63–33.94)	0.134

*Child’s educational status*						
Not yet enrolled	50	123	Reference		Reference	
Nursery	19	39	1.19 (0.62–2.27)	0.579	1.68 (0.26–10.62)	0.578
Primary	35	58	1.48 (0.87–2.53)	0.146	0.56 (0.03–9.23)	0.686
Secondary and above	16	11	3.57 (1.55–8.24)	0.003	0.66 (0.01–37.77)	0.845

*Mother’s educational status*						
Did not attend school	9	13	2.30 (0.82–6.44)	0.111	1.80 (0.07–45.76)	0.721
Primary	26	23	3.76 (1.68–8.42)	0.001	3.52 (0.32–37.94)	0.298
Secondary	70	145	1.60 (0.84–3.06)	0.147	0.29 (0.04–1.77)	0.181
University	15	50	Reference		Reference	

*Father’s occupation*						
Business	34	104	0.46 (0.22–0.96)	0.038	0.57 (0.11–3.05)	0.514
Civil servant	28	40	0.98 (0.45–2.17)	0.976	0.49 (0.06–4.23)	0.524
Private sector	41	63	0.91 (0.44–1.91)	0.821	4.49 (0.69–28.94)	0.114
Others^∗∗^	17	24	Reference		Reference	

*Size of the family*						
1–3 members	33	51	Reference			
4–6 members	46	113	0.62 (0.36–1.09)	0.102		
7+ members	41	67	0.94 (0.52–1.69)	0.852		

*Kitchen location*						
Indoor	55	120	1.93 (0.87–4.27)	0.103	10.20 (0.37–276.89)	0.167
Outdoor/separated	56	73	3.23 (1.44–7.25)	0.004	5.34 (0.21–134.96)	0.309
Both	9	38	Reference			

*Method of household waste disposal*						
Trash can in the compound	73	158	0.71 (0.45–1.13)	0.157		
Trash cash in the neighborhood	47	73	Reference			

*Main source of drinking water*						
Tap water	48	41	3.04 (1.01–9.25)	0.050		
Ground water (well)	7	10	1.82 (0.44–7.47)	0.406		
Borehole (forage)	49	133	0.95 (0.32–2.82)	0.938		
Nearby spring	11	34	0.84 (0.24–2.89)	0.784		
Mineral water	5	13	Reference			

*BCG vaccination status*						
Yes	104	223	Reference			
No	16	8	4.28 (1.77–10.33)	0.001	7.24 (1.76–18.98)	**0.041**

*Child has previous history of TB*						
Yes	50	9	17.61 (8.24–37.61)	< 0.001	20.01 (4.77–33.87)	**< 0.001**
No	70	222	Reference			

*History of TB among family members*						
Yes	71	55	4.63 (2.88–7.44)	< 0.001	4.90 (1.10–21.83)	**0.037**
No	49	176	Reference			

*Previous contact with TB patients*						
Yes	67	50	4.57 (2.83–7.37)	< 0.001	0.88 (0.04–22.18)	0.943
No	53	181	Reference			

*Duration of contact with a TB patient*						
1 week	12	19	Reference			
Few weeks	20	19	1.6 6 (0.64–4.34)	0.296	1.41 (0.31–6.43)	0.658
Few months	32	11	4.60 (1.70–12.48)	0.003	4.76 (1.02–12.14)	**0.046**

*Child’s HIV status*						
Positive	23	17	3.11 (1.17–8.22)	0.022	2.73 (1.55–19.10)	**0.032**
Negative	87	191	1.04 (0.47–2.29)	0.907	0.28 (0.02–3.42)	0.322
Unknown	10	23	Reference			

*Parent/guardian HIV status*						
Positive	23	23	1.37 (0.46–2.29)	0.563		
Negative	89	197	0.62 (0.24–1.59)	0.323		
Unknown	8	11	Reference			

*Relationship with child*						
Father	14	27	Reference			
Mother	81	151	1.03 (0.51–2.08)	0.924		
Family member	16	48	0.64 (0.27–1.51)	0.313		
Guardian	9	5	3.47 (0.97–12.35)	0.055		

*Note:* Bold values represent statistical significance.

^∗∗^Farmer, Mechanic, Journalist.

## 4. Discussion

To the best of our knowledge, this is the first study on the risk factors for childhood TB in Cameroon. This study assessed the risk factors for the occurrence of TB among children in health facilities in the Centre Region, Cameroon. A child’s previous history of TB, history of TB among family members, duration of contact with a TB patient, the absence of BCG vaccination, and the child’s HIV status were independently associated with the increased risk of childhood TB.

The findings of our study indicated that a child’s previous history of TB was one of the risk factors for childhood TB, in which the risk was higher among children who had previously been diagnosed with TB compared with children not previously diagnosed. This finding was contrary to a study conducted in Ethiopia [22], which found that previous history of TB in children was not statistically associated with TB. This difference might be due to the study location and setting, given the fact that there is an increased exposure for children living in high TB endemic communities. The results of this study suggest that children who have been previously treated for TB are more likely to develop TB again and thus underscore the need to enhance TB preventive measures.

This study found that TB disease has a fivefold increase among study participants with the history of TB among family members. In addition, children in contact with TB cases for a few months compared to 1 week were four times more likely at risk of developing TB. Several findings from Bangladesh [18, 25], Indonesia [15], Nigeria [14], Pakistan [16], and other low‐ and middle‐income settings [26] have reported similar findings. These studies found that contact history is a significant risk factor for the transmission of childhood TB. In most low‐ and middle‐income settings, even though investigation of TB contact is included in the national TB control program, it is rare and not consistently carried out due to resource limitations [26]. To improve early case detection and stop the spread of Mycobacterium TB infection, contact history investigation deserves serious consideration. Thus, contact investigation is strongly recommended, including the implementation of community‐based models of care.

Our study also revealed that children who were vaccinated for BCG had a lower risk of developing TB. This was evidenced by a meta‐analysis of available studies where the BCG vaccine can reduce the risk of TB infection by as much as 50% [27]. In addition, similar studies conducted in Ethiopia [22, 28] and India [29] reported that unvaccinated children were at a risk of developing TB. It has been hypothesized that BCG offers approximately 75% protection for 15 years when administered to children at a young age [30]. In Cameroon, the BCG coverage for children in 2022 for the Centre Region and the country was 81% and 69%, respectively, though still less than 90% as per the Global Vaccine Action Plan [31]. The Ministry of Public Health should enhance sensitization efforts regarding the BCG vaccine to address vaccine stockouts and promote vaccination during periods of availability and try community‐level efforts by healthcare workers to encourage uptake of this crucial TB vaccine for infants.

In Cameroon, TB and HIV/AIDS cannot be considered in isolation from each other. Cameroon is among the top 30 high TB‐ and TB/HIV‐burden countries and about 20% (4375) of the total number of TB cases were co‐infected with HIV in 2021; TB is the leading cause of death in HIV‐positive people, and HIV infection is a significant risk factor for TB [22, 32]. Not surprisingly, our study found that HIV‐infected children are more likely to be infected with TB.

Our study showed in the univariate analysis that young adolescents 10–14 years are at a higher risk of TB compared to children within the age group 0–4 years. This finding was supported by the Annual Report on the Implementation of Tuberculosis Control program in Cameroon that the proportion of TB patients among older children aged 5–14 years were higher than those aged 0–4 years [10]. This could be explained by the fact that as the age of the child increases, the probability of the child to spend time outside his/her home is high, which in turn increases their chance of contact with infectious cases.

In this study, several household variables such as family size, number of rooms, number of windows, kitchen location, and main source of drinking water were not related to the incidence of TB in children. Similar findings were reported by a study conducted in Indonesia [15]. This is possible because housing conditions between cases and controls did not significantly differ. However, several previous studies in South India [13], Bangladesh [25], Nigeria [14], and Ethiopia [22] stated that housing conditions were statistically associated with childhood TB.

The findings of this study should be interpreted by taking into consideration some limitations. Recall bias could be one of this study’s limitations because study participants might not be able to recall past circumstances. The controls were not sampled from the population that provided the cases. Additionally, household factors were evaluated without observation, which may have over‐ or underestimated their impact on the results. Although malnutrition is an important factor that highly contributes for the occurrence of TB in children, we were unable to analyze it due to incompleteness of most of the data for both cases and controls. Moreover, this study was observational and thus susceptible to residual confounding. In addition, the risk factors are for drug‐susceptible TB and in no way may not represent the findings for risk factors of drug‐resistant TB in children. The study strength lies in the fact that it is the first study to identify the risk factors for TB among children in Cameroon, which is critical for improved diagnosis and preventive management. Future research could be conducted at the national level with a community‐based approach, and sufficient representation of the diverse variables can help to filter out the relative effect of each factor.

## 5. Conclusion

The findings of this study have given insight into the different risk factors of childhood TB. This study showed that the absence of BCG vaccination, the child’s previous history of TB, family history with a TB patient, duration of contact with a TB patient, and child’s HIV status were identified as independent risk factors for childhood TB in the Centre Region of Cameroon. Therefore, the Centre Regional Technical Group for fight against TB is highly recommended to collaborate with various stakeholders to increase public awareness on risk factors of childhood TB and its transmission. To prevent the transmission of the infection and promote early diagnosis, the National TB Control Program should also strengthen, prioritize, and properly implement contact tracing and contact screening, including the implementation of community‐based models of care. Sensitization efforts regarding the BCG vaccine coverage in communities should be enhanced with a focus on stock management, raising awareness on the birth dose of the vaccine and integrating birth‐dose vaccination into routine maternal and newborn case services.

## Ethics Statement

This study received ethical approval from the Centre Regional Ethical Committee for Human Health Research (Ref. # CE No 031/CRERSH/2022). Administrative approval was also obtained from each of the ten selected health facilities and from the Centre Regional Delegation for Public Health (Ministry of Public Health). All parents/guardians provided written informed consent prior to study participation. Participation was voluntary as the parents/guardians were free to withdraw from the study at any time. The questionnaires contained no information that could be used to identify the respondents and were entirely anonymous.

## Disclosure

All authors read and approved the final manuscript.

## Conflicts of Interest

The authors declare no conflicts of interest.

## Author Contributions

Thomas Achombwom Vukugah contributed to the conception, design, protocol development, data collection, data analysis, and interpretation of results as well as drafting of the original draft and finalizing of the manuscript. Derick Akompab Akoku contributed to the conception, design, protocol development, drafting, and finalization of the manuscript. Micheline Mekemnang Tchoupa and Edward Lambert reviewed and provided inputs in the draft manuscript and contributed to finalizing the manuscript.

## Funding

No funding was received for this manuscript.

## Data Availability

The data that support the findings of this study are available from the corresponding author upon reasonable request.
